# Frequency analysis of mood, sleep, and alcohol and nicotine use: investigating instability in bipolar I and II disorder

**DOI:** 10.3389/fpsyt.2026.1810743

**Published:** 2026-09-03

**Authors:** Stine Holmstul Glastad, Ole Klungsøyr, Sofie Ragnhild Aminoff, Roger Hagen, Thomas Bjella, Magnus Johan Engen, Siv Hege Lyngstad, Ingrid Melle, Ole A. Andreassen, Margrethe Collier Høegh, Trine Vik Lagerberg

**Affiliations:** 1 Section for Clinical Psychosis Research, Division of Mental Health and Addiction, Oslo University Hospital, Oslo, Norway; 2 Department for Research and Innovation, Oslo Centre for Biostatistics and Epidemiology, Oslo University Hospital, Oslo, Norway; 3 Section for Early Intervention in Psychosis Advisory Service, South East Norway, Division of Mental Health and Addiction, Oslo University Hospital, Oslo, Norway; 4 Department of Psychology, University of Oslo, Oslo, Norway; 5 Department of Psychology, Norwegian University of Science and Technology, Trondheim, Norway; 6 Section for Treatment Research, Division of Mental Health and Addiction, Oslo University Hospital, Oslo, Norway; 7 Institute of Clinical Medicine, University of Oslo, Oslo, Norway; 8 Nydalen District Psychiatric Centre, Division of Mental Health and Addition, Oslo University Hospital, Oslo, Norway; 9 Center for Precision Psychiatry, Division of Mental Health and Addiction, University of Oslo and Oslo University Hospital, Oslo, Norway; 10 Department for Research and Innovation, Division of Mental Health and Addiction, Oslo University Hospital, Oslo, Norway

**Keywords:** alcohol use, bipolar disorder, instability, mood, nicotine use, sleep, time-series

## Abstract

**Background:**

Individuals with bipolar disorder (BD) often experience unstable mood beyond mood episodes, which appears to be more prominent in BD II than in BD I. It is unclear whether the mood instability and associated sleep- and substance use patterns are different between BD subtypes and also present in the first treatment phase of the disorder. In this study, we compared the degree of instability of mood, sleep characteristics, and use of nicotine and alcohol across BD I and II.

**Methods:**

We recruited 40 patients with BD in their first treatment phase (BD I n = 15, BD II n = 22 and BD Not Otherwise Specified n = 3) at Oslo University Hospital in Norway. The “MinDag” (MyDay) app was used to collect daily self-reports of mood (sadness, irritability, mood elevation and anxiety), sleep (length and quality) and weekly reports of alcohol and nicotine use for up to six months (mean no. of weeks = 18.5, a total of up to 740 weeks). To compare the degree of instability in the time-series across the two BD subtypes we used Root Mean Squared Successive Differences (rMSSD), representing the time domain, and power spectral density (PSD), representing the frequency domain. Cumulative difference periodogram (CDP) was used for group comparison.

**Results:**

Significant differences were found between BD I and BD II in the mean levels of irritability and mean hours of sleep throughout the follow-up period. The trends for higher instability in anxiety (.029) and irritability (.058) in BD II compared to BD I did not survive Bonferroni correction. Similarly, the variability of sleep length and quality and the use of nicotine and alcohol was comparable across the two groups.

**Conclusion:**

Applying longitudinal app-based self-monitoring and time-series analyses, our study did not replicate previous findings of more mood instability in BD II versus BD I. The pattern of similar instability across the groups also applied to sleep characteristics and nicotine and alcohol use. Larger samples are needed to investigate how BD subtypes can explain the instability of mood and other illness-related features, which could support the development of more personalized treatment.

## Introduction

Bipolar disorder (BD) is a severe mental disorder classified into two main subtypes: type I (BD I) and type II (BD II). The hypomanic episodes of BD II are less severe than the manic episodes of BD I (1). BD I is a disorder with distinct periods of mania and depression with periods without pronounced affective symptoms, i.e. euthymia, in between. BD II, on the other hand, has more sub-syndromal mood symptoms between episodes and shorter euthymic periods than BD I (2).

Although well established in diagnostic systems, parts of the scientific community still consider the categorization of BD into subtypes as controversial, arguing that the spectrum of BD is better captured on a continuum (3, 4). The main argument is that there appears to be few factors that distinguish one subtype from the other (3). However, studies have found differences between the subtypes in the tendency to experience mood fluctuations outside of episodes, which appears to be considerably higher in BD II compared to BD I. This has been demonstrated both for typical mood symptoms (5–7) and for affective lability (8), defined as rapid, excessive and unpredictable changes in affect (9). This is clinically important as it is associated with e.g. impaired functioning (10), increased suicidal ideation (11) and reduced life quality (12) in individuals with BD.

The introduction of smartphones and other easily accessible digital tools has made it possible to prospectively monitor mood more feasibly and validly compared to traditional clinical methods, where there is a considerable risk of recall bias and affective bias (13–15). Studies using digital tools in which patients frequently self-report their mood have indicated that BD II is more unstable than BD I with regard to depressive mood (16, 17). However, for (hypo)manic symptoms, findings are more mixed, with studies reporting no differences between BD I and II (16) and more hypomanic mood instability in adolescent with BD I compared to BD II (18). Studies investigating mood instability in BD I and II are still few, and except for the study of adolescents (18), they have mainly focused on samples with a relatively long duration of illness. The more pronounced depressive mood instability associated with BD II relative to BD I may, at least in part, be attributable to the more severe illness presentations among BD II cases that remain engaged with specialist health services over time. Thus, clarifying whether a higher degree of instability in BD II is also the case in earlier phases of the disorder, and for both depressive and (hypo)manic mood, appears warranted.

If instability of mood, i.e. a general variability of mood beyond bipolar episodes, is more characteristic of BD II than BD I, this distinction could contribute to refining the diagnostic boundaries between the two subtypes. In this context, exploring whether other features in addition to the core bipolar mood symptoms also tend to fluctuate more in BD II compared to BD I is relevant. If this is the case, assessing instability in a more general sense could facilitate the diagnostic differentiation of the two subtypes. To our knowledge, few previous studies have compared the degree of instability between BD I and II in features other than depressive and elevated mood. In the current study, we apply a broader approach and include irritability, anxiety, sleep characteristics and substance use when comparing the degree of instability between BD I and II.

With regards to anxiety, one study found differences between BD I and II in the emotional reactions after positive and adverse events. Patients with BD I reported a larger decrease in sad and anxious mood compared to controls after positive events. Patients with BD II on the other hand, experienced larger increases in anxious mood after adverse events (19). Although relevant in terms of differences in the patterns of anxious reactions to events, the study did not directly compare the degree of instability of anxious mood between the two diagnostic subtypes. With regards to irritability, again without investigating variability of irritable mood, one study found higher levels of irritable temperament in BD II patients compared to BD I (20).

Sleep disturbances are a core feature of BD illness episodes (1) and are also common in euthymia (21). Still, to our knowledge, only one previous study has compared the degree of sleep variability in BD I and II. Here, no differences were found between the subtypes in the instability of insomnia (22). However, no studies have investigated the degree of instability of other sleep features, such as sleep length- or quality in BD I relative to BD II.

In a recent study by our group, using the same sample as the current study, we applied time-series analyses to investigate temporal relationships between several mood features and alcohol- and nicotine use in participants with BD. Here, we found indications that changes in mood both preceded and were followed by changes in the levels of both nicotine and alcohol use (23). Thus, if mood instability is higher in BD II than BD I, individuals with BD II may also present with more instability in their use of nicotine and alcohol. Clarifying whether the use of nicotine and alcohol is associated with more unstable patterns in BD II compared to BD I may contribute to more targeted and personalized prevention and treatment strategies. This is an urgent clinical need as excessive alcohol and nicotine use in BD is high (24, 25) and associated with more severe clinical presentations (26–30).

In this study of individuals in their first treatment phase of BD, we compare the degree of instability in BD I and II across several mood types and other illness-relevant features. We use instability measures based on longitudinal digital self-report of sad, elevated, irritable and anxious mood, of sleep length and -quality, and of the two most frequently used substances, i.e. nicotine and alcohol. We take advantage of our frequently repeated within-subject assessment and apply statistical methods not commonly used in mental health research, but that are sensitive for detecting complex cyclical patterns and instability compared to traditional methods. We hypothesize that the degree of instability will be significantly higher in BD II relative to BD I in sad and elevated mood. For the remaining clinical features, no hypotheses are postulated as these have not been previously systematically investigated.

## Materials and methods

### Participants

This study was conducted as part of the NORMENT (Norwegian Center for Mental Disorders Research) Centre of Excellence and the Section for Clinical Psychosis Research at Oslo University Hospital (OUS) in Oslo, Norway. The participants were recruited from a catchment-area based out-patient treatment unit for BD at Nydalen District Psychiatric Center, OUS, except three participants who were included from a treatment unit for mood disorders at Haukeland University Hospital in Bergen, Norway. The specialized unit at OUS is dedicated to patients not previously diagnosed with or adequately treated for BD, and serves to initiate the first BD treatment. Inclusion criteria were age between 18 and 65 years, a diagnosis of BD type I, II or NOS (not otherwise specified), and willingness to use the “MinDag” (MyDay) app for up to six months. The exclusion criterium was lack of understanding of a Scandinavian language. The total sample comprised 40 participants: 15 with BD I, 22 with BD II and 3 with BD NOS. Participants with BD NOS had not experienced mania, but depressive and hypomanic episodes, and were therefore grouped together with BD II in all analyses and are further referred to as BD II.

The study is conducted in line with the Helsinki declaration of 1975 (as revised in 2008 and 2013) and has been approved by the Regional Committee for Medical Research Ethics and the safety representative of Oslo University Hospital. All participants provided informed consent before entering the study.

### Clinical assessments

#### Diagnostic assessments

The Structured Clinical Interview for DSM-IV axis I disorders (SCID) modules A-E or the SCID-5 Clinicians’ Version (CV) were used to conduct diagnostic evaluations. For the latter, the lifetime substance use disorder module of SCID-5 Research Version was added to DSM-5 CV as the Clinical Version only covers current use disorders (31, 32). Diagnostic evaluations were conducted by trained clinical psychologists, psychiatrists, or medical doctors, and were systematically discussed in consensus meetings with experienced clinical specialists/PhDs in psychiatry and psychology.

#### MinDag-based data

The MinDag app was developed by researchers at NORMENT in collaboration with the Center for Information Technology at the University of Oslo (33). MinDag comprises several modules where the participants rate various items either daily or weekly. The app covers several relevant features of severe mental illness, and the current study makes use of the modules for mood, sleep, and substance use. The participants were asked to use the app for six months, but shorter registration periods were accepted.

##### Mood

For the purpose of the current study, the patients were asked to answer the following question: “Please indicate to what degree the following words describe your mood today: sad, irritable, anxious, elevated/excited/on top?”. The mood module in the app is activated every evening. The items are based on an adapted version of a subscale of the Multidimensional Assessment of Thymic States (MAThyS), and are rated on a 7-point Likert scale from 1 - “not at all” to 7 – “to a large degree” (34).

##### Sleep

Sleep data was collected in the app every morning and the items in this module were based on items from the consensus sleep diary (35). Participants were asked to report the time when they went to bed, fell asleep, and woke up in the morning, and how many times they woke up during the night and for how long in total they were awake. They were also asked to indicate how well they slept on a 7-point scale from 1 – Very badly, to 7 – Very well. For the purpose of the current study, we used sleep length data based on the reported sleep onset and offset times, and self-reported sleep quality.

##### Substance use

Once a week, participants were asked to report their use of alcohol and nicotine during the last seven days. The participants were asked if they had used any nicotine, including cigarettes, e-cigarettes and the smokeless tobacco “snus” which is legal in Sweden and Norway. If yes, they were asked to report how many units of the respective nicotine type they had used on average each day. The participants could register all the different types of nicotine for each week. For alcohol, including beer, wine and liquor-based drinks, the participants were asked to report if they had used alcohol during the last seven days. If yes, they were asked how many days they had been drinking and how many units they had consumed in total.

Once a week, participants were asked to report their use of any of the following substances: hashish/marihuana, amphetamine/methamphetamine, cocaine, heroin, GHB/GBL, anabolic steroids, hallucinogens, or unprescribed medications. If they had used any of these substances in the last seven days, they were asked to report how many days they had used each substance.

##### App-based data completeness and imputation

Forty-one participants were included in the study. Participants were excluded if they had used the app for less than two weeks, which was the case for one participant, leaving a total of N = 40. The mean number of weeks of app use was 18.5, ranging from 2 to 27 (Standard deviation 7.4).

As methods for regular time-series were chosen for analysis, some data-management was necessary. To limit imputation of missing values, the daily registrations of the four mood variables and the two sleep variables were transformed into weekly means and standard deviations, based on the available data for each week. If the weekly “mean” was based on only one daily value, the standard deviations were set to zero.

Some imputation was carried out for the weekly series of alcohol and nicotine, and for one sleep value. As the number of missing weekly values was limited, we applied a simple method of imputing the mean of the preceding and the subsequent value of the time-series. In addition, the time-series was cut in the beginning and the end of the series if the participants had missing data there for either of the variables. The alcohol and nicotine use variables were only collected weekly i.e. with no weekly standard deviations, with a low number of series (as reported in **Table 1**). The imputation was performed so that all time-series analyses were applied on the same series with weekly observations to avoid irregular intervals which would include variation on arbitrary time-points. Some participants had missing data for a whole variable set. Two participants did not have data for nicotine and alcohol use and three participants did not record sleep items. These cases were thus not included in the analyses of these variables. After imputation and exclusion of the cases with missing data in a whole variable set, the total number of weeks included in the final analyses for the total sample were 740 for the mood variables, 727 for the substance use variables, and 694 for the sleep variables. For details, see **Table 1**.

**Table 1:** Information about the dataset, missing and imputation N =40.

Variables	No. of cases analyzed for rMSSD^*^/PSD	% Std = 0^**^	*% of weeks missing*	*% of weeks imputed*
Sadness	20/40	5.27% (39/740)	0	n.a.
Elevation	18/40	0.14% (1/740)	0	n.a.
Irritability	20/40	5.27% (39/740)	0	n.a.
Anxiety	19/40	5.27% (39/740)	0	n.a.
Sleep hours	16/40	6.22% (46/740)	6.35% (47^a^/740)	0.14% (1/694)
Sleep quality	20/40	6.22% (46/740)	6.35% (47^a^/740)	0.14% (1/694)
Nicotine use	10/32	n.a.	25.14% (186^b^/740)	6.10% (36/590)
Alcohol use	21/32	n.a.	25.27% (186^b^/740)	6.27% (37/590)

*Stationarity is a requirement for analyses with rMSSD. ^**^ = When the weekly “mean” was based on only one daily measure, the standard deviations were set to zero.

^a^ Three participants had no data for this variable (= 46 weeks) and were therefore not included in the analyses.

^b^ Two participants had no data for both nicotine and alcohol use (= 13 weeks) and six participants had time-series where missingness was considered too high for imputation (= 173 weeks). These eight participants were not included in the analyses.

### Statistical analyses

The statistical analyses were conducted with the use of IBM Statistical Package for Social Sciences version 29 and R. SPSS was used for the descriptive analyses and bivariate analyses. Bivariate analyses were conducted with Chi square tests or Mann Whitney U tests as applicable. These tests were two-tailed and the significance level set at 0.05. R was used for time-series analyses, as described below.

**Figure 1:**
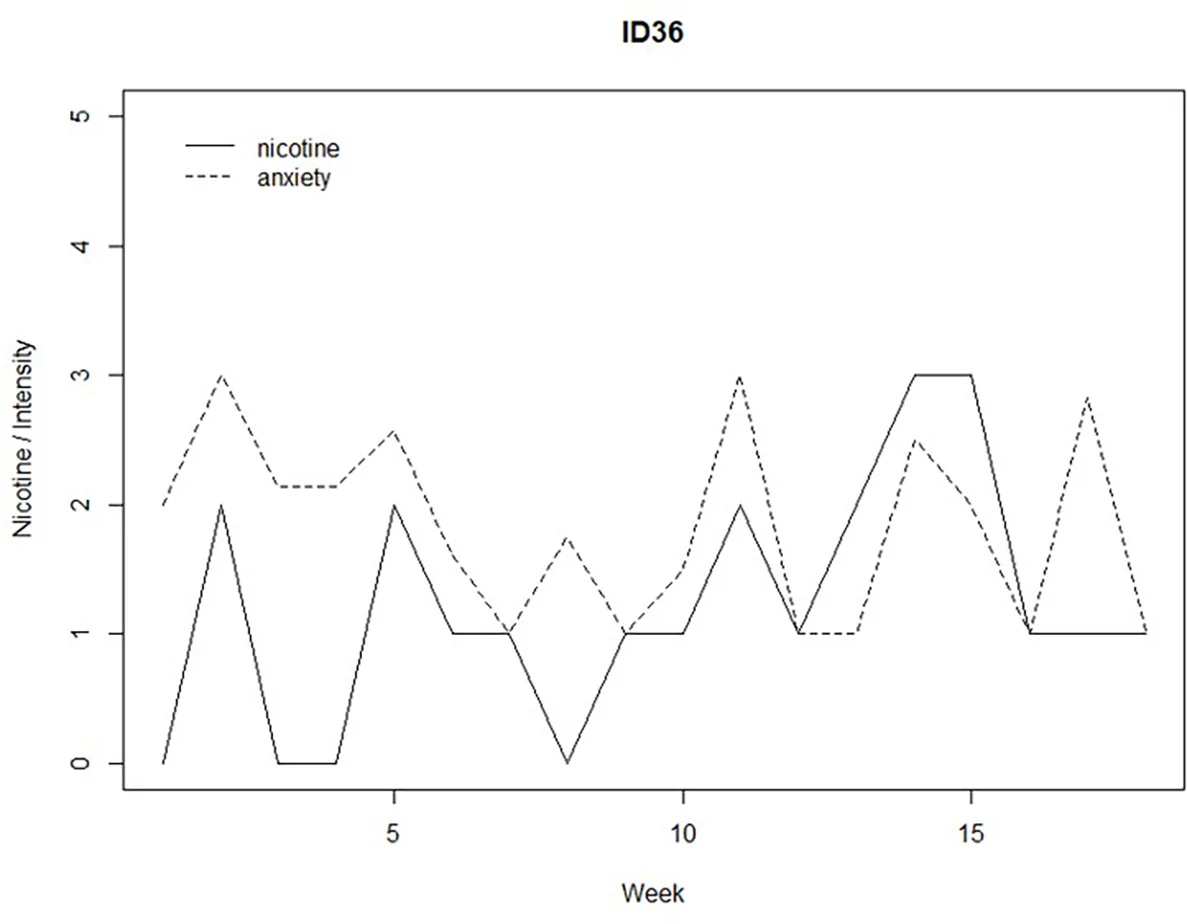
Time series of nicotine units per week and anxiety for patient ID=36.

The total dataset comprised 14 time-series in total, one per variable: weekly mean levels of four mood categories; sadness, mood elevation, irritability and anxiety with corresponding standard deviations, weekly mean hours and quality of sleep with corresponding standard deviations, and weekly mean units of alcohol and nicotine consumed. Examples of raw time-series data is shown in **Figure 1** for illustration.

Time-series are sufficiently represented in either time- or frequency domains. Different qualities are elucidated in the two domains, with the autocovariance function of a stationary series as a building block in both. The autocovariance expresses dependence across different lags in the series, the “memory” of the process, and can be used to understand how much one value is dependent on previous values in the series. In the time-domain this is used directly for modeling and forecasting. The frequency domain analysis is based on the Fourier-transformed autocovariance function, the so-called power spectral density (PSD) and describes the content of the series in terms of amount of energy (power) in all series as a function of frequency (36), which frequencies contain the most energy. The pragmatic decision to analyze all data on a weekly level implies a sampling interval of 1 week. This had the important implication that signals with shorter periods than two weeks could not be studied, except indirectly through the standard deviations. The frequency is given as number of cycles per sampling interval (alternative to radians per sampling interval). A maximum frequency of 0.5 (the Nyquist rate, please see Appendix for further description) therefore corresponds to a cycle of two weeks. The spectral density is symmetric around zero (cycle of infinite length), so it suffices to study the interval from 0 to 0.5.

### Frequency domain representation

The basic estimator for the PSD is the periodogram (Appendix). Assessment of group differences relies on an aggregated effect-measure. The average of the raw time-series at each time-point is not informative due to the nature of individual processes, but PSD is comparable across series. A group mean PSD (periodogram) describes the average amplitude of cyclic components within the group for a range of frequencies. If the PSD is dominated by high frequencies, it means that the time-series is more erratic and consists of periodic signals of shorter cycles. Frequencies near 0 describe a constant and slowly varying signal. The highest frequency when observations are registered weekly is a signal with cycle of two weeks (Nyquist rate) (37). The *spectrum* command in the STATS package in R is used for analysis (38).

### Time domain representation

The Root Mean Squared Successive Difference (rMSSD) was used as a time domain measure to compare dynamics in time-series in line with previous literature on instability-related phenomena (Appendix) (39–42). It captures both within person variability and temporal dependence (first order). The rMSSD is relatively robust for temporal trends and extensions to unbalanced designs (different number of observations and lengths of intervals between them within persons (41) and is also informative for non-stationary time-series. The rMSSD is univariate (a scalar).

### Group comparison

To compare BD I and BD II with respect to their frequency profiles, permutation tests were performed (43). Both univariate tests for all variables separately and a multivariate combination test across different categories were of interest. To correct for multiple testing, we divided a significance level of 0.05 by the number of tests within each category (Bonferroni adjustment), e.g., for mood, the reference level was 0.05/8 = 0.006.

For the frequency domain, the cumulative absolute value of the difference between the logarithm of the mean of the periodogram for the two groups was constructed, called “cumulative difference periodogram” (*CDP*_v_) (Appendix). The group sizes varied across the 14 different variables due to partial missingness for some series. To assess the magnitude of the group difference, an approximate effect-size was estimated for the *CDP*_v_ by dividing the magnitude on a bootstrap-estimate of the standard-error (signal-to-noise ratio). A Cohen’s d – like effect-size estimate was calculated by interpreting the signal-to-noise ratio as a z-score and adjusting for sample-size. For a more extensive description of the statistical procedures, see Appendix.

In the time domain, the group difference was assessed by the absolute value of the difference in logarithm of the mean rMSSD in the two groups (Appendix).

## Results

### Demographics and clinical characteristics

The sample consisted of 40 participants, 24 female (60%), with a mean age of 31.8 years. Seven participants (17%) had a comorbid substance use disorder, and 8 participants (20%) had a comorbid anxiety disorder. There were significant differences between BD I and BD II throughout the follow-up period in the mean levels of irritability (p = .026), and in the mean number of hours slept each week (p = .013). Demographic and clinical characteristics of the sample are presented in **Table 2**.

**Table 2:** Demographic and clinical characteristics of the sample (N = 40).

*Characteristics*	*BD I* *(n=15)*	*BD II* *(n=25)*	*Total*	p
Female sex, n (%)	7 (46.7)	17 (68)	24 (60)	.317
Age, years, median (IQR) Min - max, years	29.00 (19) 22 - 57	29.00 (12) 18 - 52	29.00 (14) 18 – 57	.619
Comorbid substance use disorders, n (%) Alcohol use disorder, current, n (%) Alcohol use disorder, lifetime (%) Illicit substance use disorder^a^, current, n (%) Illicit substance use disorder^a^, lifetime, n (%)	2 (5) 1 (2.5) 0 (0) 0 (0) 1 (2.5)	5 (20) 1 (4) 3 (12) 1 (4) 1 (4)	7 (17.5) 2 (5) 3 (7.5) 1 (2.5) 2 (5)	.691
Comorbid anxiety disorders^b^, n (%)	2 (13.3)	6 (24)	8 (20)	.686
Nicotine use, n (%) Nicotine units per week^c^, median (IQR) Min - max, weekly use	8^f^ (57.14) 10.00 (10.25) 0 - 45	13^f^ (54.17) 12.00 (16.00) 0 - 130	21 (55.3) 10.00 (15.00) 0 -130	.750
Alcohol use, n (%) Alcohol units per week^c, d^, median (IQR) Min - max, weekly use	14^f^ (100) 3.00 (6.00) 0 - 44	24^f^ (100) 6.00 (10.00) 0 - 43	38 (100) 5.00 (10.00) 0 – 44	.106
Any illicit substance use^e^, n (%)	0	5 (20)	5 (12.5)	
Mood Elevated mood, median (IQR) Irritability, median (IQR) Sadness, median (IQR) Anxiety, median (IQR)	1.29 (2.00) 1.14 (1.00) 1.33 (1.5) 1.50 (1.86)	1.50 (1.50) 1.80 (1.36) 2.00 (1.7) 1.80 (2.00)	1.43 (1.70) 1.50 (1.40) 2.00 (1.8) 1.67 (2.00)	.783 .026* .119 .543
Hours of sleep, median (IQR) Min – max, hours	7.67^f^ (1.46) 4 - 13	7.17^g^ (1.50) 3.33 – 9.71	7.33 (1.38) 3.33 - 13	.013*
Quality of sleep, median (IQR)	4.00^f^ (1.94)	4.57^g^ (1.57)	4.33 (1.61)	.200

*BD* = Bipolar Disorder*, IQR* = Interquartile Range.

The range of mood variable and quality of sleep scores was from 1 to 7.

The median number of units, range and the analyses are listed only for the participants who reported use of the substances.

*= p <.05.

^a^ Includes the following disorders: Cannabis, Cocaine, MDMA and Hallucinogens.

^b^ Includes the following disorders: Social Phobia, Generalized Anxiety Disorder, Panic Disorder, Agoraphobia and Post Traumatic Stress Disorder.

^c^ n=2 missing for both nicotine and alcohol use and n=6 had time-series where missingness was considered too high for imputation. These 8 cases excluded from the analyses.

^d^ The square root was used for analyses.

^e^ Includes the following substances: Cocaine, MDMA, Amphetamine and Cannabis.

^f^ n=1 missing.

^g^ n=2 missing.

### Time-series descriptions

**Figure 2:**
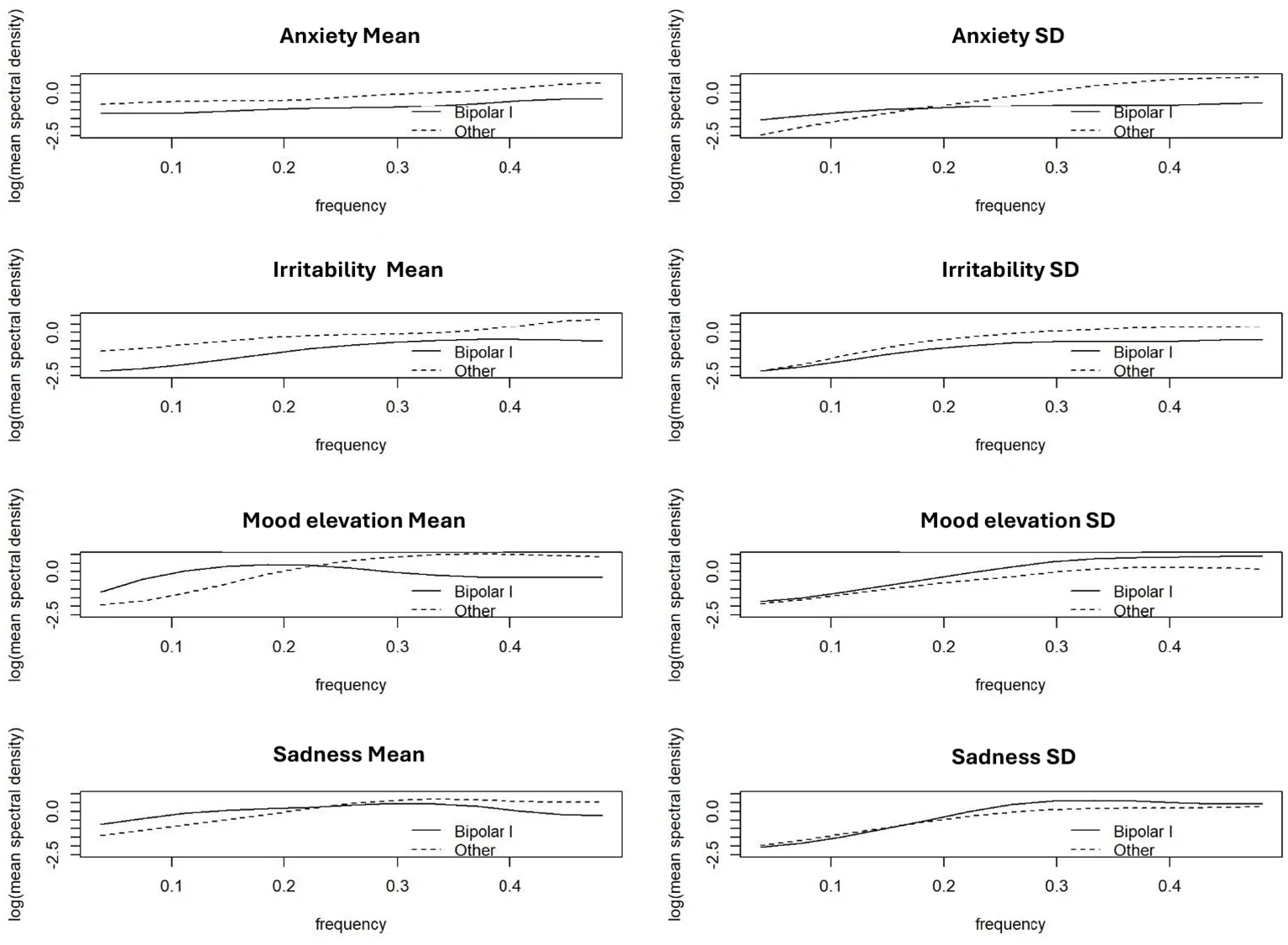
Mean log-spectra for BD I and Other (BD II + BD not otherwise specified) from time-series of weekly mean and standard deviation (SD) for mood-variables (anxiety, irritability, elevation and sadness). The x-axis shows frequency in cycles per week. A frequency of 0.5 cycles/week corresponds to a period of 2 weeks, 0.25 cycles/week to 4 weeks, and lower frequencies correspond to longer-term fluctuations (e.g. 0.05 cycles/week = 20-week period).

In general, the spectra, i.e. cyclical behavior and frequency components, of all the 14 time-series were dominated by high frequencies. Namely, the most important cyclic components in the series had cycles from 2 weeks (shortest possible cycle) to 3 months (midway between shortest and longest cycle), for sadness, anxiety and irritability, except mood elevation in the BD I group. None of the categories had significant differences between the BD I and BD II groups in the multivariate tests (please refer to further descriptions below), however, in the mood category, the amplitudes were smaller in the BD I group for the dominating frequencies in six of the eight variables (combined p = 0.103) (**Figure 2** and **Table 3**). The Cohen d-like effect sizes of the *CDP_v_* ranged from small (0.32) to very large (1.44).

**Table 3:** Permutation test from and (and approximate effect-sizes) for time-series of weekly mood, sleep, mean alcohol consumption (sqrt), nicotine units, and a multivariate combination test.

Variables	*p-value, ΔrMSSD_v_*	*No. of series for BD I/ BD II for rMSSD*	*p-value CDP_v_*	*Nr. of stationary series for BD I/ BD II for CDP_v_*	Effect-size, CDP*_v_*
Mood					
Anxiety, mean Anxiety, Std Irritability, mean Irritability, Std Mood elevation, mean Mood elevation, Std Sadness, mean Sadness, Std Combined	0.51 0.41 0.40 0.08 0.44 0.65 0.16 0.12 0.22	15/25 15/25 15/25 15/25 15/25 15/25 15/25 15/25	0.15 0.03 0.06 0.25 0.76 0.41 0.52 0.62 0.10	11/14 8/16 11/14 7/14 5/15 7/14 10/14 8/17	0.79 1.44 0.94 0.66 1.13 0.90 0.68 0.47
Sleep					
Sleep hours, mean Sleep hours, Std Sleep quality, mean Sleep quality, Std Combined	0.17 0.87 0.65 0.95 0.75	15/25 15/25 15/25 15/25	0.05 0.72 0.60 0.26 0.25	10/14 8/12 9/17 7/15	1.30 0.50 0.59 0.80
Substance use					
Alcohol (sqrt) Nicotine Combined	0.87 0.93 0.98	12/20 12/20	0.74 0.83 0.94	7/14 4/6	0.32 1.17

### Mood

The number of stationary time-series in the BD I and II groups for the different mood variables is shown in **Table 3**. None of the univariate tests of differences between the BD I and II groups yielded statistically significant results when adjusted for multiple testing, with the largest difference for weekly standard deviation in anxiety (p = 0.029) for the *CDP*_v_ test with the BD I group having numerically smaller amplitudes in the dominating frequency range (short cycles), i.e. less variation in the weekly standard deviation for short-term fluctuations, compared to the BD II group (**Figure 2**). The same tendency was found for weekly mean irritability (p = 0.058). The combined test showed an indication of a difference in mood for *CDP*_v_ (p = 0.103), but not for Δ*rMSSD*_v_ (p = 0.223), with the BD I group having smaller amplitudes for the dominating frequencies in most dimensions.

### Sleep

**Figure 3:**
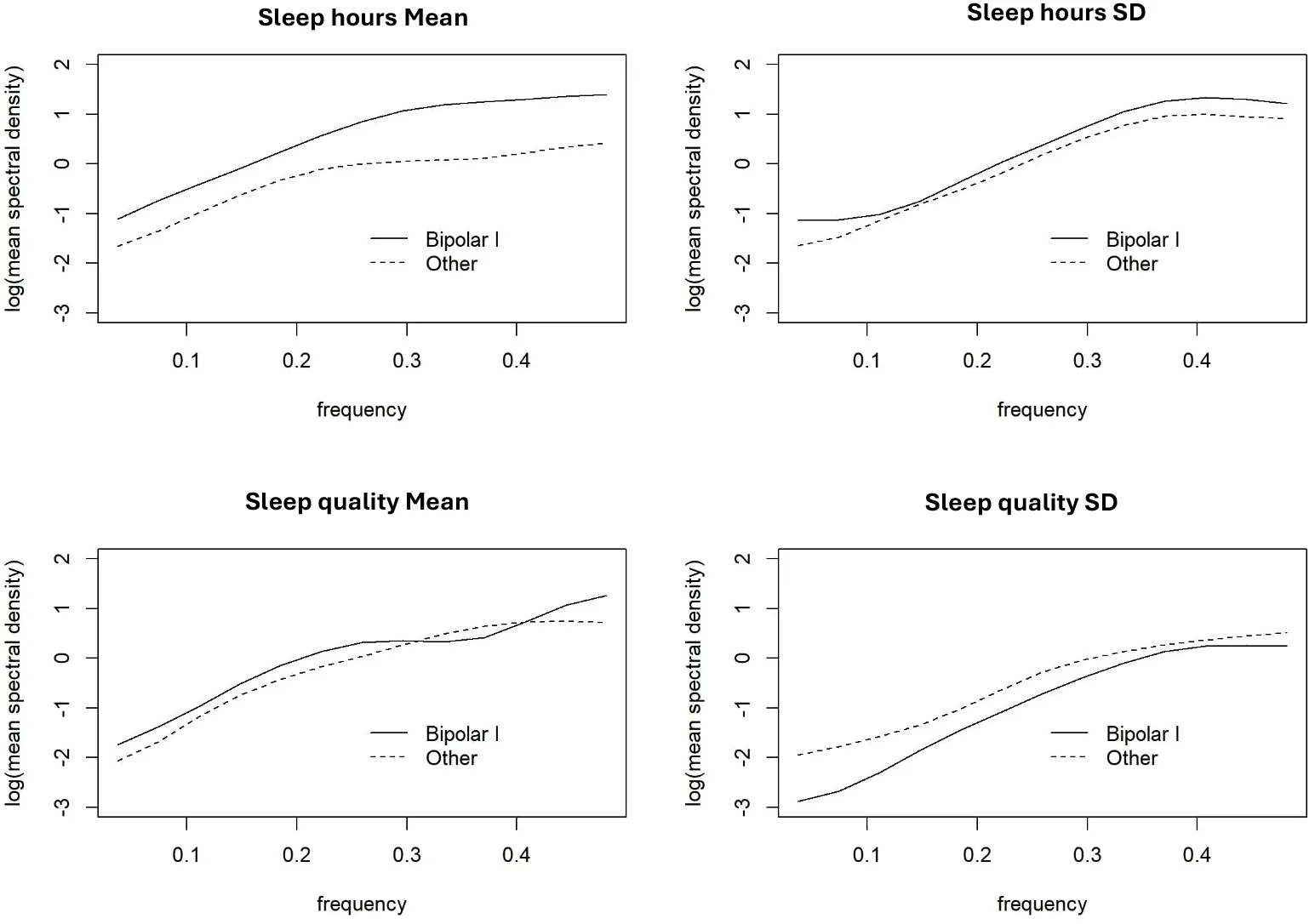
Mean log-spectra for Bipolar I and Other (BD II + BD not otherwise specified) from time-series of weekly mean and standard deviation (SD) for sleep assessments (hours and quality). The x-axis shows frequency in cycles per week. A frequency of 0.5 cycles/week corresponds to a period of 2 weeks, 0.25 cycles/week to 4 weeks, and lower frequencies correspond to longer-term fluctuations (e.g. 0.05 cycles/week = 20-week period).

None of the univariate tests of differences in the Sleep category between the BD I and II groups yielded statistically significant results with or without adjustment for multiple testing. The largest difference was found for mean number of hours sleep (p = 0.052, **Table 3**) where the BD I group had larger amplitudes for the dominating frequencies (higher frequencies) than the BD II group (**Figure 3**). This indicated that the BD I group showed more variation in their sleep patterns compared to the BD II group (*CDP*_v_ p = 0.052 vs. Δ*rMSSD*_v_ p = 0.168, respectively) although this was not statistically significant. The combined tests did not show any significant difference between the groups, p = 0.248 vs. p = 0.753, again the *CDP*_v_ was most powerful (**Table 3**).

### Nicotine and alcohol

**Figure 4:**
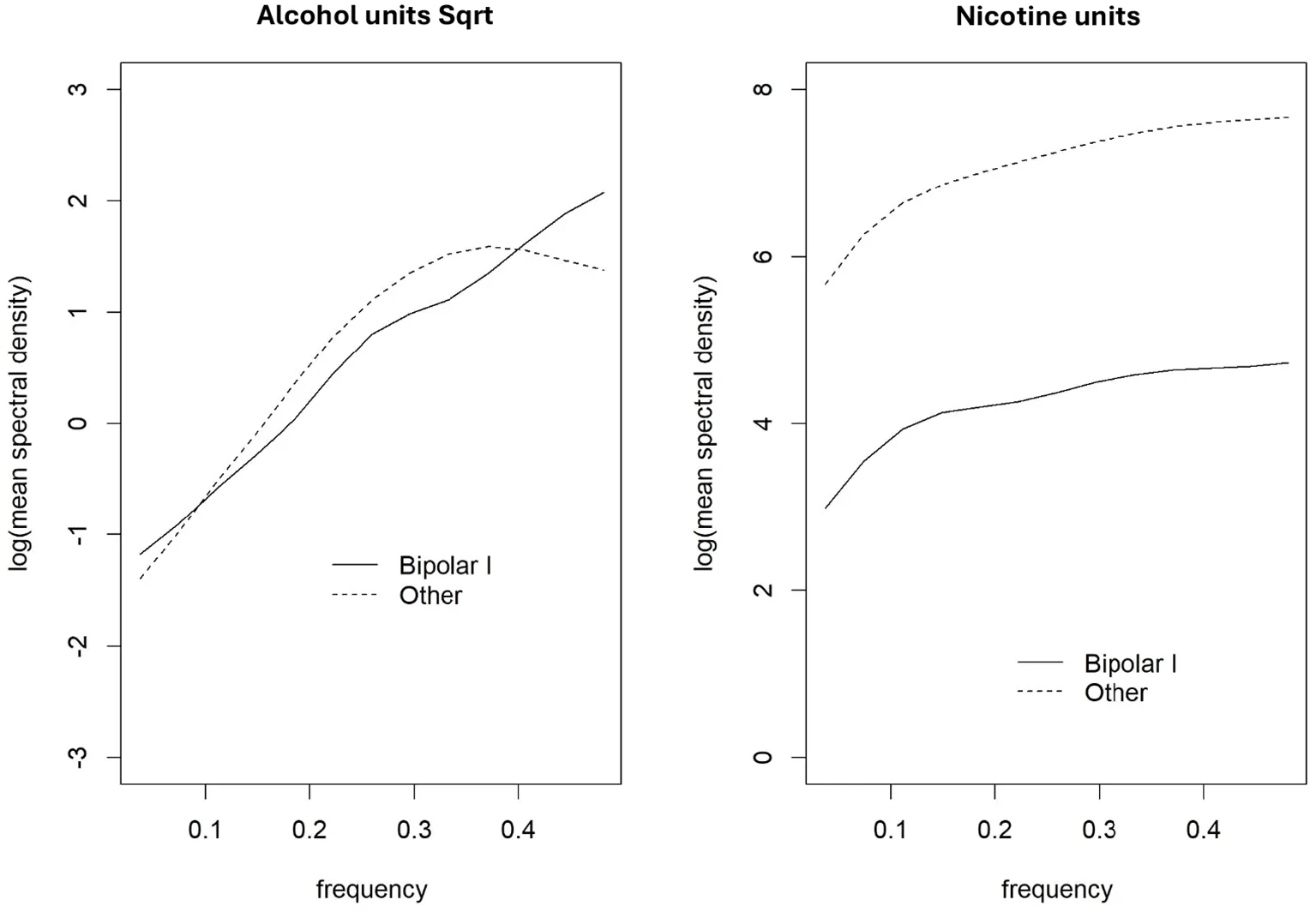
Mean log-spectra for Bipolar I and Other (BD II + BD not otherwise specified) from time-series of weekly mean alcohol consumption (sqrt) and nicotine units. The x-axis shows frequency in cycles per week. A frequency of 0.5 cycles/week corresponds to a period of 2 weeks, 0.25 cycles/week to 4 weeks, and lower frequencies correspond to longer-term fluctuations (e.g. 0.05 cycles/week = 20-week period).

For weekly reported alcohol units (square root transformed) and units of nicotine, the number of series was limited due to non-stationarity and missing observations. For alcohol the diagnostic groups had similar spectra whereas for nicotine the BD I group had much smaller mean spectral density for all frequencies. However, with only 10 stationary time-series for nicotine use and some outliers, the uncertainty was considerable (**Figure 4**). As shown in **Table 3**, neither the univariate tests or the combination tests showed any sign of differences, with p = 0.939 for *CDP*_v_ and p = 0.982 for Δ*rMSSD*_v_. For the latter, non-stationary series were not excluded, but despite a larger sample size and more precision for estimating the difference in means, no significant difference was found.

## Discussion

In this study, we investigated time-series data to assess putative differences between BD I and BD II in the degree of instability in several mood and sleep features, and for the use of nicotine and alcohol. Both the Root Mean Squared Successive Differences (rMSSD), representing the time-domain, and the power spectral density (PSD), representing the frequency domain were used to operationalize differences. The results did not show evidence of significant differences in the time-series between the two diagnostic groups in mood elevation, irritability, sadness, anxiety, in the number of hours of sleep or sleep quality, or in the use of nicotine or alcohol.

Although the test combining all mood categories showed a trend toward larger amplitudes in BD II than in BD I, we did not observe clear support for our hypothesis that individuals with BD II would present with more mood instability than individuals with BD I. However, the modest size of the study limits the strength of this conclusion. Our finding of equivocal instability of depressed mood in the BD subtypes diverges from previous studies. This includes a few that, similarly to our own, utilized frequent longitudinal measures and reported higher instability in BD II compared to BD I. For (hypo)manic symptoms, however, previous findings have been mixed (16, 18). To our knowledge, the degree of instability of other mood features such as irritability and anxiety has not been compared across BD I and II in previous studies. In the present study, BD II showed numerically higher amplitudes for the dominating frequencies in both the weekly means and the standard deviations of anxiety and irritability compared to BD I. However, these differences did not remain statistically significant when adjusting for multiple testing.

Several factors may account for the lack of significant differences in the degree of mood instability between BD I and II, although caution should be taken in the interpretation due to the relatively small sample size and potential uncontrolled confounders. Firstly, the participants were recruited from a specialized clinical unit for patients without a previous history of adequate treatment of BD, which represents a sample relatively early in the illness course. While mood instability may be similar in the two BD subtypes in the early phases of the disorder, the higher instability seen in more chronic BD II samples could stem from a sampling bias, as patients with persistent mood instability may be more likely to remain engaged with psychiatric care. Nonetheless, such an interpretation remains somewhat speculative and warrants further investigation. Furthermore, one may also speculate that the current findings are related to different treatment responses in the two BD subtypes. Although pharmacological treatment appears to be equally efficient in BD I and II (44), there are indications from some studies that individuals with BD II have a slower response compared to BD I (45), which may also apply to depressive mood instability. Furthermore, pharmacological treatment could influence both average mood and mood variability, and unmeasured treatment changes may therefore have impacted the instability patterns. Unfortunately, information about pharmacological treatment was not systematically recorded throughout the current study’s follow-up period. Such treatment could thus have affected the diagnostic groups differently, potentially confounding our results.

Secondly, the contrast to previous studies may be related to methodology, i.e. operationalization of clinical phenomena, data collection methods and/or statistical methods. In the current study, we focused on mood instability, operationalized as week-by-week fluctuations in self-reported mood. This is different from e.g. affective lability, which refers to a general experience of being prone to short-lived and rapid switches of affective states. Furthermore, our approach of computing weekly means based on daily ratings, may have yielded results that differ from previous studies (16, 17). However, we consider this as unlikely since we also investigated potential differences in the time-series of the standard deviations, which capture the day-to-day variability inherent in our data.

A last possible interpretation of the lack of differences could be a “masking” effect of different symptomatic states in BD I and II during the study period, e.g. that the proportion of participants who experienced affective episodes were higher in BD I than in BD II. Information about syndromal depressive and (hypo)manic status was not systematically collected, as our aim was to characterize overall mood variability irrespective of episodes. We also regard mood variability across affective states over time as an important feature of the illness course in and of itself. However, as mood variability can differ between affective episodes and euthymic periods, information about affective states during the follow-up period would have been of importance, as it may have influenced the results and masked the lack of difference found between the two groups.

In the current study, we found that the variability of sleep length and -quality was similar in the two groups, except for a tendency for less instability (amplitude) in BD II for weekly mean hours of sleep. Thus, at least in the first treatment phases of the disorder, sleep instability appears to be similar across the BD subtypes. Given the limited research exploring potential differences between BD I and BD II in different sleep parameters, further studies are needed to corroborate this finding.

Similarly, no significant differences were found in the variability of nicotine- and alcohol use between the two diagnostic subtypes. As there were no differences in mood instability between the two groups and given that changes in nicotine and alcohol use appear to accompany mood changes (23), this finding may not be surprising.

Previous studies investigating mood instability in BD with high-resolution longitudinal data have mainly applied the Root Mean Square Successive Difference method (rMSSD) (e.g.^46^). We ran both rMSSD and PSD frequency analyses. The latter may be considered unconventional for this type of data. As the PSD analyses yielded lower p-values for most univariate tests and for all combination tests as compared with the rMSSD, the PSD comparison may represent a more powerful test, although caution should be taken as no formal comparison of the two tests was conducted. Although rMSSD incorporates both variation and first-order temporal dependence, higher-order temporal dependence is not covered, and a change in magnitude of rMSSD does not identify whether it is due to a change in variance or the opposite change in correlation, or a combination. In the high dimensional PSD, more variation will result in higher amplitudes in the underlying cyclic components (higher PSD), and less correlation will be represented by higher PSD for higher frequencies relative to lower frequencies. Limitations in rMSSD have recently been described in a recent heart rate variability study (47), which leaves frequency analysis as an alternative. Effect-sizes were all non-negligible although bias was likely (skewness in the bootstrap distributions and small sample-sizes). This indicates that low power is a plausible explanation for lack of more group differences.

In the context of current findings, the debate concerning the categorization of BD into discrete subgroups as opposed to conceptualizing the disorder along a continuum (4) is relevant. Our results indicate that the instability of mood and other illness-related features is similar across BD I and BD II, showing no significant divergence between the two diagnostic groups. However, we consider the current results as inconclusive regarding the continuum hypothesis, as there is still need for further research on larger samples that also incorporate information about affective episodes and pharmacological treatment during the follow-up period.

### Limitations and strengths

The study has several limitations. The sample size was relatively small. Also, the aggregation of daily ratings of mood and sleep variables into weekly means may have caused a loss in variability signals. However, standard deviations as an expression of variability within each week were also compared between the groups. To avoid exclusion of relatively intact time-series, imputation was performed for the alcohol and nicotine variables. Imputation of values based on the mean from adjacent timepoints in the nicotine and alcohol use variables, may have introduced bias in the form of reduced variability. However, as the imputation performed was relatively limited (6.3% at most), we regard this risk as modest. Furthermore, the degree of missing data was comparable to other similar studies using app-based self-report (48). Also, relevant information about the participants’ previous illness history, affective episodes and pharmacological treatment throughout the follow-up period is lacking. Lastly, the BD II group included three participants with BD NOS leaving a non-uniform diagnostic group. Still, we consider it unlikely that this has masked underlying differences between the two groups that were compared in the analyses.

The study also has several strengths. As recruitment to the study was conducted mainly from a catchment area-based treatment unit, the sample is likely to be representative for patients in the first treatment of BD. We collected longitudinal intensive app-based data for several BD-relevant parameters simultaneously. Furthermore, the permutation test applied has clear advantages as it is a non–parametric test with no distributional assumptions. The combination of several univariate tests into one multivariate test is straight forward, with the dependence between the univariate tests intact and without the need for an explicit model for this dependence. For example, in the mood domain such dependence gave an indication of a group difference in the multivariate test although the differences were less prominent in the univariate tests. In addition, with easy access to computational power, a high number of repeated random draws without replacement from the data ensures asymptotic results with high accuracy. Moreover, although explorative in the current study, using a spectral approach may have several benefits, including the identification of between-group effects at different frequencies.

## Conclusion

In this study of participants in their first treatment of BD I and II, we collected longitudinal app-based data of sad, elevated, irritable and anxious mood, sleep features and the use of nicotine and alcohol. We applied sensitive time-series analyses, which yielded no significant differences in instability between participants with BD I and BD II in any of these parameters. This is in contrast to previous findings from more chronic BD samples. Although we did not observe the expected effects, our work demonstrates the feasibility of applying frequency analyses to psychiatric time-series and suggests that such methods merit further use. Due to the inconclusive results from the current study, further research exploring time-series of BD-relevant features across the subtypes is needed.

## Data Availability

The raw data supporting the conclusions of this article will be made available by the authors, without undue reservation.
