# Supplementary material for: Frequency analysis of mood, sleep, and alcohol and nicotine use: investigating instability in bipolar I and II disorder
Source: Front Psychiatry. 2026 Sep 3;17:1810743. doi: 10.3389/fpsyt.2026.1810743 (PMC13582255; doi:10.3389/fpsyt.2026.1810743)
Supplement: Supplementary file 1 [file SupplementaryFile1.docx]

**Appendix**

***Time-series representation in the frequency domain***

The basic estimator for the PSD is the periodogram, denoted by $I_{i}\left( \omega\right)$. The smoothed version of the 14 different series in the present dataset is denoted $\tilde{I_{v,i}}\left( \omega\right)$, for person $i=1,\cdots,N$ and variable $v=1,\cdots,14$. Assessment of group differences relies on an aggregated effect-measure. The average of the raw time-series at each time-point is not informative due to the nature of individual processes, but PSD is comparable across series. A group mean PSD (periodogram) describes the average amplitude of cyclic components within the group for a range of frequencies. The PSD carries a large amount of information, due to its high dimension (a continuous function of frequency).

Analysis of time-series in the frequency domain is particularly well suited for revealing presence and distribution of periodicities. The power spectral density (PSD) express power (energy) in the series as a function of frequency. The PSD for a strictly cyclic signal/series will have a discrete spectrum with spikes corresponding to the different frequency components, whereas a non-periodic series will have a continuous spectrum. The most used estimator for PSD is the periodogram, given by

$I_{i}\left( \omega\right)=\frac{1}{K}\left\{ \left[ \sum_{k=1}^{K} Y_{i,k}sin\left( \omega k \right) \right]^{2}+\left[ \sum_{k=1}^{K} Y_{i,k}cos\left( \omega k \right) \right]^{2} \right\}$	(A1)

a squared correlation between the series $Y_{i,k}$ for person $i=1,\cdots,N$ at time-points $k=1,\cdots,K$ and the sine/cosine waves of frequency $\omega$. Consistency is achieved by means of smoothing, and a logarithm – transformation has a variance stabilizing effect (variance independent of the spectral magnitude) (Venables & Ripley, 2013). The upper limit of the frequency in the periodogram corresponds to 0.5 cycles between time–points which in the present data is a cycle of two weeks (observations every week) (Nyquist rate) (Priestley, 1981). The spectral density is symmetric around 0, so positive frequencies between 0 and 0.5 constitutes a sufficient representation.

Smoothing of the periodogram is necessary to make the estimator consistent. The amount of smoothing is recommended based on trial- and error (Venables & Ripley, 2013). Sufficient smoothing will preserve major features like spikes in the PSD and remove random fluctuations. The “spectrum” function in R uses repeated moving averages by modified Daniell smoothers (Bloomfield, 2000), specified by the user. In our case two Daniell smoothers were applied (spans(3,5)). The results were not sensitive to degree of smoothing, because extra smoothing was performed in the effect-measure anyway, by averaging over persons within group for every frequency.

Series of different lengths needed individual fitting to ensure that the same frequency components were assessed for each series. This was accomplished by prolonging each series with zeros on each side (padding) until the length is appropriate for the Fast Fourier Transform to automatically assess the desired frequencies (make the length equal to a “highly composite number”).

***Time domain representation***

It captures both within person variability and temporal dependence (first order) and can be written as

$r{MSSD}_{v,i}=\frac{1}{K-1}\sum_{k=2}^{K} \left( Y_{v,i,k}-Y_{v,i,k-1} \right)^{2}$	(1)

where $Y_{v,i,k}$ is the $k$th time-point of the observed time-series for person $i=1,\cdots,N$ and variable $v=1,\cdots,14$.

***Permutation test***

A multivariate permutation test was performed, one for each of the following three categories: alcohol and nicotine with two dimensions ($d=2$), sleep with four dimensions ($d=4$): weekly mean number of hours, weekly standard deviation in number of hours, weekly mean quality of sleep, weekly standard deviation in quality of sleep, and mood with eight dimensions ($d=8$): weekly mean and standard deviations in anxiety, irritability, elevation and sadness. The multivariate version of the test statistics for no difference between the groups (with all variables within a category considered simultaneously) and with the dependence between the univariate tests preserved, was based on Fisher combination function (Pesarin & Salmaso, 2010).

The permutation test is a flexible computer–based test that produces exact p–values (conditional on the data) with several strengths.

A test in the frequency domain can be constructed in a number of ways, for example to highlight a specific range of frequencies or to put more weight on strata (or frequencies) with less variance. Here, the cumulative absolute value of the difference between the logarithm of the mean of the periodogram for the two groups was constructed, called “cumulative difference periodogram” (${CDP}_{v}$). All frequencies are equally weighted, and the test statistic is given by

${CDP}_{v}=\sum_{f=1}^{F} abs\left( log\left( \frac{1}{N_{0}}\sum_{grp=0,i} \tilde{I_{v,i}}\left( \omega_{f} \right) \right)-log\left( \frac{1}{N_{1}}\sum_{grp=1,i} \tilde{I_{v,i}}\left( \omega_{f} \right) \right) \right)$	(2)

where the $\omega_{f}$, $f=1,\cdots,F$ are the different frequencies over the range $\left( 0,0.5 \right)$. Here, $F=13$ was found appropriate (varies with amount of smoothing). $Grp=0$ corresponds to the BD I group, and the $abs\left( . \right)$ means that a difference in either direction will contribute equally as will crossing vs no crossing PSDs (could be replaced by $\left( . \right)^{2}$)

To compare the groups based on the time domain measure rMSSD, a simple group average difference (without sign) was assessed, given by

${\Delta rMSSD}_{v}=\sqrt{abs\left( log\left( \frac{1}{N_{0}}\sum_{grp=0,i} r{MSSD}_{v,i} \right)-log\left( \frac{1}{N_{1}}\sum_{grp=1,i} {rMSSD}_{v,i} \right) \right)}$	(3)

The group sizes $N_{0},N_{1}$ varied across the 14 different variables due to partial missingness for some series. The permutation test was based on $N=N_{0}+N_{1}$ random draws without replacement from the observed group categories with the group sizes preserved. The permuted group assignment together with the rest of the dataset unchanged constituted one permuted dataset. This was stratified by the permuted group assignment and the test-statistics ${CDP}_{v}$ and ${\Delta rMSSD}_{v}$ were calculated. With these steps repeated a large number of times the null distribution was formed (distribution under the null hypothesis of no difference between the groups) for which the observed test-statistics were compared to give permutation test p-values (Table 1 in Appendix). The test-statistics (denoted by $T_{v}$) were calculated for each variable and permutation. With “*” as the permuted group assignment, $T_{v=1}^{*,1}, \cdots,T_{v=14}^{*,1}$ denotes the 14 different variables for the first permutation. With the observed test-statistic (the $T_{v}$ for the observed group categories) denoted by $T_{v}^{obs}$ the “observed” p-value is given by

$p_{v}^{obs}={\#\left\{ T_{v}^{*,perm}\geq T_{v}^{obs} \right\}}/M, perm=1,\cdots,M$	(4)

where $\#\left\{ . \right\}$ indicates number of times the statement in the parentheses is true when $perm$ varies, and $M$ is a large number (e.g. 1000).

The multivariate version of the test statistics for no difference between the groups (with all variables within a category considered simultaneously) and with the dependence between the univariate tests preserved, was based on Fisher combination function (Pesarin & Salmaso, 2010), given by

$T_{comb}^{obs}=-\sum_{v=1}^{d} log\left( p_{v}^{obs} \right)$	(5)

(an alternative combination was also tested with negligible difference). No extra permutations were necessary. The existing permutations were used to find the null distribution for $T_{comb}^{obs}$ simply by calculating for each permutation, a permutation p-value (one for each variable in the category), in the same way as $p_{v}^{obs}$ from (4) was calculated (Table 1 in Appendix). The collection of permuted combination test-statistics formed the null distribution for which $T_{comb}^{obs}$ was compared to give the overall p-value for the combination test.

The permutation test is a flexible computer–based test that produces exact p–values (conditional on the data) with several strengths. The p-value in (4) is derived by the following algorithm:

1 Calculate on the given data set $\boldsymbol{Y=}\left\{ {grp}_{i},Y_{v,i}, i=1,\cdots,N, v=1,\cdots,14 \right\}$ the observed value of the test-statistic $T_{v}^{obs}$ ($Y_{v,i}$ is either the periodogram – a vector of length = 13 or the scalar $r{MSSD}_{v,i}$, for person $i$ and variable $v$, and $T_{v}^{obs}$ is either ${CDP}_{v}^{obs}$or ${\Delta rMSSD}_{v}^{obs}$)
2 Take a random permutation of the data $\boldsymbol{Y}^{\boldsymbol{*}}$, obtained by considering a random permutation of the group assignments together with the original $Y_{v,i}'s$
3 Calculate $T_{v}^{*}$
4 Independently repeat steps 2 and 3 $M$ times

The *p*-value is calculated by equation (4)

***Table 1****: Permutation test construction*

| $T_{1}^{*,1}, p_{1}^{*,1}$ | $\cdots$ | $T_{d}^{*,1}, p_{d}^{*,1}$ | $T_{comb}^{*,1}$ |
| --- | --- | --- | --- |
| $\vdots$ |  | $\vdots$ | $\vdots$ |
| $T_{1}^{*, M}, p_{1}^{*, M}$ | $\cdots$ | $T_{d}^{*, M}, p_{d}^{*, M}$ | $T_{comb}^{*, M}$ |
| $T_{1}^{obs}, p_{1}^{obs}$ | $\cdots$ | $T_{d}^{obs}, p_{d}^{obs}$ | $T_{comb}^{obs}$ |

**References**

Bloomfield, P. (2000). *Fourier analysis of time series: an introduction*. New York: John Wiley & Sons.

Pesarin, F., & Salmaso, L. (2010). *Permutation tests for complex data: theory, applications and software*. Chichester, West Sussex, UK: John Wiley & Sons.

Priestley, M. B. (1981). *Spectral analysis and time series. Probability and mathematical statistics*. London: Academic Press.

Venables, W. N., & Ripley, B. D. (2013). *Modern applied statistics with S*. New York: Springer Science & Business Media.
